# The Extent and Predictors of Fathers’ Involvement in the Raising of Children with Autism Spectrum Disorder in the UAE

**DOI:** 10.3390/ijerph22020300

**Published:** 2025-02-17

**Authors:** Maxwell Opoku, Ahmed Mohamed, Shamsa Almarzooqi, Aisha Cheikhmous

**Affiliations:** 1 Department of Special and Gifted Education, College of Education, United Arab Emirates University, Al Ain P.O. Box 15551, United Arab Emirates; maxwell.p@uaeu.ac.ae (M.O.); 201280183@uaeu.ac.ae (A.C.); 2 Zayed Higher Organization for People of Determination, Abu Dhabi 15551, United Arab Emirates; 200908775@uaeu.ac.ae

**Keywords:** fathers, involvement, children, autism spectrum disorder, United Arab Emirates

## Abstract

Background: Raising children with autism spectrum disorder (ASD) has consistently been found to be challenging for parents. However, available studies have mainly focused on mothers, raising questions about fathers’ involvement in raising children with ASD. Indeed, fathers’ involvement has consistently been reported as fundamental to the development of children with ASD. Thus, it necessitates extensions of Western-dominated literature to novel contexts such as the United Arab Emirates (UAE). The purpose of this study, therefore, was to explore the extent and predictors of fathers’ participation in the nurturance of children living with ASD. Methods: The survey was completed by 177 fathers raising children with ASD in the UAE and was based on the revised 27-item Fathers’ Involvement in Development and Rehabilitation Scale. The Statistical Package for Social Science was used to compute means and perform a multivariate analysis of variance and hierarchical multiple regression. Results: Fathers’ ratings were high on involvement: attitude, support, and participation in training to assist their children with ASD. Also, support and participation in training significantly contributed to the variance in attitude towards children with ASD. Moreover, the place of residence of participants made a significant contribution to the variance in attitude towards children with ASD. Conclusions: There is a need for policymakers to capitalize on the gains already made in creating a favorable environment for the development of children with ASD in the UAE. Regular engagement between policymakers and fathers could enhance their engagement in raising their children.

## 1. Introduction

Community-based disability rehabilitation has been advocated by scholars as fundamental in the effort towards promoting the well-being of children with disabilities [1,2,3]. The rehabilitation process encompasses the utilization of existing community services or resources, such as schools and hospitals, to assist in the development of children with disabilities [2]. However, the first step towards promoting the participation of children with disabilities in community services is to have a supportive family who would facilitate their participation [4,5,6]. At the micro-level, both parents of a child with disabilities are expected to be involved in nurturing them and promoting their participation in essential services within the community [7,8]. However, in the non-Western context, children with disabilities, such as those living with autism spectrum disorder (ASD), continue to struggle to access social amenities such as education within society [9,10,11,12,13,14,15]. With the voices of mothers consistently being captured in research [13,14,15,16], there is an urgent need to explore paternal involvement in the raising of children with ASD.

In this study, involvement was defined as behavioral (support and training) and affective (attitudinal) relationships between parents and their children with ASD. Understandably, studies on parental involvement in the raising of children are gaining traction based on the proposition that parental involvement has a positive impact on developmental domains such as intelligence, social, and physical development [17,18,19,20,21,22]. The development is more pronounced in the event that fathers are involved in the raising of their children, including those with ASD [23,24,25]. This has ignited discussions on the need for systems to encourage fathers to contribute meaningfully when it comes to the raising of children with ASD [26]. While the challenges that mothers have in raising children with ASD have been researched and reported on [13,14,15,16], the voices of fathers are somehow conspicuously missing in the non-Western literature.

Previous research has attempted to study fathers’ involvement in the raising of children with ASD [17,18,20,21,22,26,27,28,29,30,31,32,33,34,35,36]. These studies could be broadly categorized into three areas: experiences [18,27,30], support [34,35], and challenges raising children with ASD [20,21,22,28,33]. In terms of experiences, fathers accept children with ASD as integral members of the nuclear family [18,26]. Similarly, some of the research studies showed that fathers assist their children with schoolwork and facilitate their participation in society [34,35]. Nonetheless, inasmuch as fathers appreciate their children as equal members of society, they encounter some challenges, such as stress, financial problems, and less time to support their children with ASD [18,30,36]. These studies are limited in scope because such exploration is yet to be extended to non-Western contexts such as the Unted Arab Emirates.

ASD, as defined by [37], is a neurodevelopmental disorder with deficits in three qualitative areas that guided this study: communication, repetitive behavior, and social functioning. Out of the over 5000 children with disabilities enrolled in school in the UAE, nearly half are living with ASD [38]. It is estimated that 1 out of every 146 children is living with ASD in the UAE—an estimate projected to grow due to environment-related factors [39]. However, the onset of ASD is linked to the superstitious belief that ASD is caused by evil persons within society [9,10,13,40]. Furthermore, children with ASD are discriminated against in communities and face formidable barriers when it comes to accessing essential services within society [11,12,40]. In this specific cultural context, it is useful to study fathers’ involvement in the raising of children with ASD.

Two objectives guided this study. First, this study aimed at exploring the extent of fathers’ participation in the development of children with ASD. Another aim was to explore the relationship between the affective and behavioral involvement of fathers in the raising of children with ASD. The study questions were:

1. How do fathers rate their involvement in the nurturance of their children who have been diagnosed with ASD?
2. Which personal profile could provide an additional explanation of fathers’ involvement?
3. Will behavioral involvement contribute to the variance in the affective involvement of fathers?

## 2. Methods

The current study formed part of a large project that aimed to develop an in-depth understanding of fathers’ role in the development of children with disabilities [41]. The current study drew on fathers to develop insight into fathers’ contributions towards raising children who have been diagnosed with ASD.

### 2.1. Study Participants

This study drew on fathers raising children with ASD in the UAE which consists of seven states (emirates): Abu Dhabi, Ajman, Umm Al Quwain, Ras Al Khaimah, Fujairah Dubai, and Sharjah. The UAE is an Islamic republic with Arabic mainly spoken in the country. However, national development is aligned towards modern infrastructural development, technological innovation, and promoting the local culture. Out of the estimated 9.7 million people, over 80% are made up of expats working in the oil and service industries.

The inclusion criteria were as follows: (a) fathers with children with ASD; (b) children formally diagnosed with ASD and enrolled in school; (c) at least 18 years old; (d) ability to read in either English or Arabic; and (e) Consenting to participate in this study.

One hundred and seventy-seven fathers with children with ASD were recruited nationally for this study (see Table 1).

### 2.2. Instrument

A two-part instrument was used to collect data from fathers. The first included background data about fathers (see Table 1 for details).

The second part was the revised 27-item Fathers’ Involvement in Development and Rehabilitation Scale (FIDRS; see Table 2), which was developed for this study to assess paternal involvement in raising children with disabilities [41]. The instrument was developed based on a thorough literature review [17,20,21,22,31,33,34,35] on each of the tenets that informed the development of the items. It has been found to have appropriate psychometric properties [41].

The instrument included three domains. The first domain is attitude towards parenting which is made up of nine items and further sub-divided into two sub-scales, beliefs towards support and parenting. The second domain is support which is also made up of 14 items and sub-divided into three sub-scales, namely well-being, personal support, and participation in learning. The third domain is participation in training which is a unidimensional scale.

The instrument was subjected to content and statistical validation before being used for this study. For this study, calculation of the scale’s reliability using Cronbach’s Alpha yielded appropriate values: support (0.88), attitude (0.91), and training (0.94).

### 2.3. Procedure

The Social Sciences Ethics Review Committee at United Arab Emirates University (ERSC_2023_2467) approved this study and its protocols. Following this, permission was sought from governmental bodies (Emirates Schools Establishment (currently Ministry of Education), Ministry of Community Development, and Zayed Higher Organization for People of Determination) to collect data from schools. Special schools were contacted to obtain permission to conduct this study. The organizations/centers that agreed to participate were sent the survey link. The funding organization of this study sent text messages to parents of children with disabilities in Abu Dhabi and that was the largest tactic to recruit many parents to participate in this research study.

The QuestionPro platform was used to disseminate the survey. The data were collected in two languages, Arabic and English, which enabled participants to complete the survey in their language of proficiency. The data collection lasted for five months, from February 2023 to June 2023. The survey included an information statement that explained the study’s purposes and significance for the UAE context. The participants were informed that their identity would be kept confidential. All the participants signed a written consent before completing the survey.

### 2.4. Data Analysis

Microsoft Excel was used to clean the data, and then they were imported to SPSS version 29 for statistical analysis. The data were normally distributed and, as such, suitable for a parametric test.

Thereafter, the researchers answered the research questions. To answer research question 1, means were computed to develop insights into fathers’ involvement.

In relation to research question 2, multivariate analysis of variance (MANOVA) was performed to ascertain the differences between participants [6]. The assumptions of linearity, outliers, and homogeneity of variance were not violated. A Bonferroni-adjusted alpha of 0.01 [6] was adopted. An effect size (partial eta squared) was used and interpreted as follows: small (0.01–0.05), moderate (0.06–0.1), and large (at least 0.1) [6].

For research question 3, hierarchical multiple regression was computed. Prior to the computation, Pearson’s correlation was examined to understand the relationship between the continuous variables: attitude, support, and participation in training. For the multiple regression, attitude was operationalized as an outcome variable, and support and training were used as predictors while controlling for demographic variables. Assumptions of normality, linearity, multicollinearity, and homoscedasticity were not violated [6].

## 3. Results

### 3.1. Level of Fathers’ Involvement

The main scores were as follows: support for children with ASD (M = 4.58, SD = 0.42), attitude towards children with ASD (M = 4.23, SD = 0.78), and participation in training (M = 4.00, SD = 0.50).

### 3.2. Association Between Demographic Variables and Involvement

MANOVAs were computed to explore the association between demographic variables and involvement (see Table 2). First, there was a difference between nationality in the combined dependent variables, F (3, 173) = 3.87, Wilks’ Lambda = 0.94, *p* = 0.01, and partial eta squared = 0.06. On the domains, a difference was observed in attitudes towards children with ASD only, F (1, 175) = 9.73, *p* = 0.002, and partial eta squared = 0.05. The mean scores showed that citizens of the UAE (M = 4.40, SD = 0.62) who had children with ASD demonstrated more favorable attitudes than expats (M = 4.04, SD = 0.89).

Second, the difference between participants in the combined dependent variables in residence was significant: F (3, 172) = 4.92, Wilks’ Lambda = 0.85, *p* = 0.001, and partial eta squared = 0.08. Individually, a difference was found between participants in attitudes, F (1, 177) = 6.07, *p* = 0.001, and partial eta squared = 0.07. A Tukey’s HSD post hoc comparison test showed that those in the Northern Emirates (M = 4.67, SD = 0.39) differed from those living in Dubai (M = 4.10, SD = 0.36). However, both did not differ from those living in Abu Dhabi (M = 4.14, SD = 0.84).

### 3.3. Support and Training as Predictors of Attitude

The calculation of the correlation co-efficient yielded the following: support and participation in training (*r* = 0.14, *p* = 0.06), support and attitude (*r* = 0.32, *p* = 0.001), and attitude and participation in training (*r* = 0.26, *p* = 0.001).

Multiple Linear Regression was computed to understand the impact of support and participation in training on the variance in attitudes towards children with ASD while controlling for demographic variables (see Table 3 below). In step 1, support (beta = 0.28, *p* = 0.001) and training (beta = 0.22, *p* = 0.003) made a significant contribution of 15% in the variance in attitude, F (2, 169) = 14.33, *p* = 0.001.

Step 2 included adding demographic variables to the model to explore their influence on the model. The demographics made an only 8% insignificant contribution, F (11, 158) = 1.70, *p* = 0.08. However, the combined demographics and the two predictors made a 24% significant contribution to the variance in attitudes, F (13, 171) = 3.74, *p* = 0.001. Additionally, while the two continuous variables (support and training) made a significant contribution, among the demographic variables, only the place of residence of participants significantly contributed to the variance in attitude towards children with ASD.

## 4. Discussion

The study reported here was conducted due to the lack of literature on fathers’ involvement in the lives of their children with ASD. Most importantly, the results showed that fathers rated themselves highly on each of the tenets of involvement. This finding slightly agrees with previous Western studies which reported that fathers held a favorable disposition and were committed to supporting their children with ASD [18,27,30]. The findings reported in this study are promising and demonstrate that fathers of children with communication, behavioral, and social deficits related to ASD self-report that they are involved in their upbringing. This result could be credited to the steps taken by the UAE’s government to bring disability issues to the mainstream, develop policies, and advocate for the inclusion of children with ASD in society [11,12]. While cultural stereotypes about the onset of ASD are rife in communities [9,10,13,40], fathers appear to demonstrate commitment towards nurturing their children. The findings reported here are promising and could be consolidated or capitalized on by policymakers through regular engagement with fathers to understand their needs and take steps to address them.

In the UAE, anecdotal evidence showed persistent and deep-seated negativity towards children with ASD, which derails the development of children with ASD [9,10,13]. In this study, the computation of multiple regression showed that support and participation in training made a significant contribution to the variance in the attitudes of fathers towards children with ASD. This trend identified here is expected because communities harbor negative feelings toward children with disabilities, which contributes to familial tensions [13]. This probably suggests that changing the attitudes of fathers has the potential to impact the support they offer to their children with ASD. It is thus recommended that policymakers engage in conversations with fathers about how to embrace their children with ASD and offer them training in ways to support their development.

The nationality of fathers provided further understanding of their involvement in the raising of their children. The results showed that fathers who are citizens of the UAE rated themselves more positively than expat fathers raising children with ASD. It is useful to mention that expats constitute over 80% of the national population. Moreover, the expats have traveled to the UAE to work and might have a small social network to rely on for emotional or psychological support. This may indicate that expat parents in the UAE are struggling to raise children with ASD in the UAE [13]. However, it could be inferred that citizens of the UAE live close to their immediate and extended family members, who in turn might offer social support when needed. There is, therefore, the need for policymakers to tailor training programs or intervention support to suit the uniqueness of expat fathers in the UAE.

Participants also differed in residence. While the MANOVA showed that fathers in the Northern Emirates indicated more favorable attitudes than those in Abu Dhabi, the regression showed that fathers in Abu Dhabi are 1.6 times more likely to demonstrate positive attitudes towards children with ASD than those living in the Northern Emirates. A previous study in the UAE reported differences in stress levels related to raising children with disabilities between parents based on their place of residence [16]. Although it is difficult to explain the rationale behind this result, the contextual arrangement could offer some clues on what seems to be happening within the country. The UAE has different services or supervisory bodies based on the jurisdiction where fathers live. The disparities between MANOVA and regression, as well as contextual peculiarity, lend support for future studies to delve deep into the paternal experiences of involvement in the raising of children with ASD.

This study had some limitations. First, the study participants included children with ASD in special schools or rehabilitation centers. The fathers yet to enroll their children with ASD in schools were not considered for participation. The findings cannot be extrapolated to fathers whose children are out of school. However, fathers who participated in this study and those who were excluded share common access to support systems and services. The study findings could reflect the experiences of fathers who were excluded from this study. Second, fathers’ self-reported experiences guided this study; thus, their responses might be biased. Moreover, this study did not intend to verify participants’ reported claims. An information statement was provided to fathers, and they rated their involvement in their preferred language. It is possible that they reported accurate accounts of their involvement. Regardless of this, a future study could compare the ratings given by both fathers and mothers regarding the former’s role in the nurturing of children with ASD. Furthermore, future studies could use qualitative methods where fathers would be asked to describe their experiences and the extent of their involvement in the lives of their children. More so, future studies could involve professionals such as teachers and health workers to understand their perspectives on fathers’ involvement in the development of children with ASD.

## 5. Conclusions

The study reported here assessed the extent of fathers’ involvement in the raising of children with ASD in the UAE. The study drew on fathers who were raising children with ASD to understand their attitudes, support they provide to their children and participation in training to contribute towards the raising of their children. The results showed high ratings of fathers on attitudes, support and participation in training to contribute towards the development of their children with ASD. Additionally, support and participation in training emerged as predictors of fathers’ attitudes towards their children. Moreover, demographic variables such as place of residence, fathers’ nationality provided additional insight into involvement in the raising of children with ASD in the UAE.

The current study is novel as it has added to the literature, the extent of fathers’ involvement in the development of their children with ASD. The study has also provided important guidelines which could be considered in future policy reforms in the UAE. For instance, changing attitudes of fathers towards children with ASD maybe a dependent on fathers support at home as well as participation in training. As part of effort towards changing attitudes, policymakers could train fathers on ways through which they could support their children at home as well as educating them about training opportunities which fathers could participate to learn about parenting strategies for children with ASD. Moreover, there could be regular interaction with fathers to understand their concerns, needs and support services that are available to their children with ASD. This could enable fathers to be aware of useful services in the communities, enable them to make inputs and be active in the raising of children with ASD. All children need both parents in their lives and some of the suggestions above could be implemented by policymakers to enhance the development of children with ASD in the UAE.

## Figures and Tables

**Table 1:** Summary of participants’ demographic characteristics.

Categories (*n* = 177)	Frequency	Percentage
Age (in years)		
20–30	7	4%
31–40	78	44%
41–50	71	40%
51 and above	21	12%
Nationality		
Citizen	92	52%
Expats	85	48%
Residence		
Abu Dhabi	136	77%
Dubai	11	6%
Northern Emirates	41	17%
Education		
At most Secondary	71	40%
Bachelor	74	42%
Graduate	31	18%
Employment (n = 176)		
Unemployed	19	11%
Employed	158	89%
Monthly family income		
Less than 10,000	50	28%
10,000–20,000	40	23%
21,000–30,000	39	22%
31,000 or more	48	27%
Years of marriage (n = 174; in years)		
Less than 10	143	82%
11–20	25	14%
31 or more	6	4%
Children’s gender		
Male	132	75%
Female	45	25%
Children’s age (n = 176)		
1–8 years	133	76%
9–13 years	27	15%
14 years or more	16	9%
Severity of disability		
Mild	36	20%
Moderate	117	66%
Severe	24	14%
School enrolment		
Yes	131	74%
No	46	26%

Calculation of the scale’s reliability using Cronbach’s Alpha yielded the following results: support (0.88), attitude (0.91), and training (0.94).

**Table 2:** Summary of multivariate analysis of variance.

Categories	Wilks’ Lambda	MAN. F	ANOVA F.		
			Support	Attitude	Training
Age	0.94				
F		1.30	2.20	0.23	1.04
partial eta squared		0.02	0.04	0.004	0.02
Nationality	0.94				
F		3.87 **	0.14	9.73 **	3.62
partial eta squared		0.06	0.001	0.05	0.02
Residence	0.85				
F		4.92 **	9.71 **	6.07 **	1.16
partial eta squared		0.08	0.10	0.07	0.01
Education	0.96				
F		1.12	0.05	2.48	0.97
partial eta squared		0.02	0.001	0.03	0.01
Employment level	0.99				
F		0.56	0.04	0.01	1.49
partial eta squared		0.01	0.001	0.001	0.008
Monthly family income	0.93				
F		1.46	1.80	3.54	0.28
partial eta squared		0.03	0.03	0.06	0.005
Years of marriage	0.97				
F		0.88	0.65	0.56	1.13
partial eta squared		0.02	0.008	0.006	0.01
Children’s gender	0.98				
F		1.08	0.58	0.16	2.19
partial eta squared		0.02	0.003	0.001	0.01
Children’s age	0.94				
F		1.74	1.64	1.32	1.13
partial eta squared		0.03	0.02	0.02	0.01
Severity of disability	0.97				
F		0.77	0.42	0.86	1.32
partial eta squared		0.01	0.005	0.01	0.02
School enrolment	0.98				
F		1.11	2.16	0.71	0.46
partial eta squared		0.02	0.01	0.004	0.003

** *p* ≤ 0.01; partial eta squared = effect size.

**Table 3:** Support and training regressed on attitude towards children with ASD.

Model	Uns. B	S.E.	Stan. Beta	t	*p*
Step 1					
Support	0.34	0.09	0.28	3.95	0.001 **
Training	0.77	0.25	0.22	3.03	0.003 **
Step 2					
Support	0.31	0.09	0.26	3.59	0.001 **
Training	0.60	0.26	0.17	2.33	0.02 *
Age	0.30	0.74	0.03	0.40	0.69
Nationality	−0.78	1.30	−0.06	−0.60	0.55
Residence	1.58	0.73	0.17	2.15	0.03 *
Education	−1.47	0.77	−0.15	−1.90	0.06
Employment	1.27	1.77	0.05	0.71	0.48
Family income	0.67	0.54	0.11	1.23	0.22
Years of marriage	−0.53	1.15	−0.04	−0.46	0.65
Gender of children	−0.60	1.17	−0.04	−0.51	0.61
Age of children	0.72	0.84	0.07	0.85	0.40
Severity	−0.55	0.89	−0.05	−0.62	0.53
School enrolment	−0.79	1.08	−0.06	−0.73	0.47

** *p* ≤ 0.01; * *p* ≤ 0.05.; Uns. B = Standard Beta; S.E. = Standard Beta; Stan. Beta = Standardized Beta.

## Data Availability

The original contributions presented in this study are included in the article. Further inquiries can be directed to the corresponding author(s).
